# Supplementary material for: Heat adaptation of phage T7 under an extended genetic code
Source: Virus Evol. 2021 Dec 1;7(2):veab100. doi: 10.1093/ve/veab100 (PMC8923235; doi:10.1093/ve/veab100)
Supplement: veab100_Supp [file veab100_supp.zip › Sequencing_Analysis.zip!/Sequencing_Analysis/S1/evidence/SNP_7.html]

BRESEQ :: Evidence


| Predicted mutation | | | | | | |
| --- | --- | --- | --- | --- | --- | --- |
| evidence | seq id | position | mutation | annotation | gene | description |
| RA | NC\_001604.1 | 26,756 | A→G | intergenic (–/–) | – / – | –/– |

| Read alignment evidence... | | | | | | | | | | | |
| --- | --- | --- | --- | --- | --- | --- | --- | --- | --- | --- | --- |
|  | seq id | position | | ref | new | freq | score (cons/poly) | reads | annotation | genes | product |
| \* | NC\_001604.1 | 26,756 | 0 | A | G | 96.9% | 11285.0 / 113.3 | 3998 | intergenic (–/–) | *–/–* | –/– |
| Reads supporting (aligned to +/- strand):  **ref** base A (57/66);  **new** base G (1897/1918);  **total** (1992/2006) | | | | | | | | | | | |
| Fisher's exact test for biased strand distribution *p*-value = 4.65e-01 | | | | | | | | | | | |
| Kolmogorov-Smirnov test that lower quality scores support variant *p*-value = 8.31e-01 | | | | | | | | | | | |

Only 100 of 4008 total aligned reads displayed.  

`GTGGTCTCATTGGGACTTTGGGGAAAACGTACAGGTTCTAGCTTGTCAGAGTATCAGCTCAGATATGTATGTGATTCTTCGCAATGAGTTCAATACGTTCCTAGCTAGAATCTCTTTCACTAAGAACGCCATTGACTTACAGGGAGAACCCTATCGTGCCTTTATGGACATGAAGATTCGATACACGATTCCTAGTGGAACATACAACGATGACACATTCACTACCTCTATTCATATTCCAACAATTTATGGTGCAAACTTCGGGAGGGGCAAAATCACTGTATTGGAGCCTGATGGTAAGATAACCGTGTTTGAGCAACCTACGGCTGGGTGGAATAGCGACCCTTGGCTGAGACTCAGCGGTAACTTGGAGGGACGCATGGTGTACATTGGGTTCAACATTAACTTCGTATATGAGTTCTCTAAGTTCCTCATCAAGCAGACTGCCGACGACGGGTCTACCTCCACGGAAGACATTGGGCGCTTACAGTTACGCCGAGCGTGGGTTAACTACGAGAACTCTGGTACGTTTGACATTTATGTTGAGAACCAATCGTCTAACTGGAAGTACACAATGGCTGGTGCCCGATTAGGCTC  >  NC_001604.1/26458‑27054`  

                                                                                                                                                                                                                                                                                                          `|`                                                                                                                                                                                                                                                                                                            
`tttataagtgacagccTTTGGGGAAAACGGACAGGTTCTAGCTTGTCAGCGTATCAGCTCAGATATGTATGTGATTCTTCGCAATGAGTTCAATACGTTCCTAGCTAGAATCTCTTTCACTAAGAACGCCATTGACTTACAGGGAGAACCCTATCGTGCCTTTATGGACATGAAGATTCGATACATGATTCCTAGTGGAACATACAACGATGACACATTCACTACCTCTATTCATATTCCAACAATTTATGGTGCAAACTTCGGGAGGGGCAAAATCACTGTATTGGAGCCTGATGGTGAg                                                                                                                                                                                                                                                                                                          <  1:493462/286‑1 (MQ=255)`  
       `cATTGGGACTTTGGGGAAAACGTACAGGTTCTAGCTTGTCAGAGTATCAGCTCAGATATGTATGTGATTCTTCGCAATGAGTTCAATACGTTCCTAGCTAGAATCTCTTTCACTAAGAACGCCATTGACTTACAGGGAGAAACCTATCGTGCCTTTATGGACATGAAGATTCGATACACGATTCCTAGTGTAACATACAACGATGACACATTCACTACCTCTATTCATATTCCAACAATTTATGGTTCAAACTTCGGGAGGGGCAAAATCACTGTATTGGAACCTGATGGTGCAATAACCg                                                                                                                                                                                                                                                                                                   >  1:391938/1‑301 (MQ=255)`  
                          `aCGTACAGGTTCTAGCTTGTCAGAGTATCAGCTCAGATATGTATGTGATTCTTCGCAATGAGTTCAATACGTTCCTAGCTAGAATCTCTTTCACTAAGAACGCCATTGACTTACAGGGAGAACCCTATCGTGCCTTTATGGACATGAAGATTCGATACACGATTCCTAGTGGAACATACAACGATGACACATTCACTACCTCTATTCATATTCCAACAATTTATGGTGCAAACTTCGGGAGGGGCAAAATCACTGTATTGGAGCCTGATGGTGAGATAACCGTGTTTGAGCAACCTACGGc                                                                                                                                                                                                                                                                                <  1:132621/301‑1 (MQ=255)`  
                          `aCGTACAGGTTCTAGCTTGTCAGAGTATCAGCTCAGATATGTATGTGATTCTTCGCAATGAGTTCAATACGTTCCTAGCTAGAATCTCTTTCACTAAGAACGCCATTGACTTACAGGGAGAACCCTATCGTGCCTTTATGGACATGAAGATTCGATACACGATTCCTAGTGGAACATACAACGATGACACATTCACTACCTCTATTCATATTCCAACAATTTATGGTGCAAACTTCGGGAGGGGCAAAATCACTGTATTGGAGCCTGATGGTGAGATAACCGTGCTTGAGCAACCTACGGc                                                                                                                                                                                                                                                                                >  1:144705/1‑301 (MQ=255)`  
                                  `gTTCTAGCTTGTCAGAGTATCAGCTCAGATATGTATGTGATTCTTCGCAATGAGTTCAATACGTTCCTAGCTAGAATCTCTTTCACTAAGAACGCCATTGACTTACAGGGAGAACCCTATCGTGCCTTTATGGACATGAAGATTCGATACACGATTCCTAGTGGAACATACAACGATGACACATTCACTACCTCTCTTCATATTCCACCAATTTATGGTGCAAATTTCGGGAGGGGCAAAATCACTGTATTGGAGCCTGATGGTGAGATAACCGTGTTTGAGCAACCTACGGCTGGGTGGa                                                                                                                                                                                                                                                                        >  1:481695/1‑301 (MQ=255)`  
                                      `tgggctcggagatgtgtataagagacaggtatgtGATTCTTCGCAATGAGTTCAATACGTTCCTAGCTAGAATCTCTTTCACTAAGAACGCCATTGACTTACAGGGAGAACCCTATCGTACCTTTAAGGACATGAAGATTCGATACACGATTCCTAGTGGAACATACAACGATGACACATTCACTACCTCTATTCATATTCCAACAATTTATGGTGCAAACTTCGGGAGGGGCAAAATCACTGTATTGGAGCCTGATGGTGAGATAACCGTGTTTGAGCAACCTACGGCTGGGTGGAATAg                                                                                                                                                                                                                                                                    <  1:604112/273‑1 (MQ=255)`  
                                            `gTCAGAGTATCAGCTCAGATATGTATGTGATTCTTCGCAATGAGTTCAATACGTTCCTAGCTAGAATCTCTTTCACTAAGAACGCCATTGACTTACAGGGAGAACCCTATCGTGCCTTTATGGACATGAAGATTCGATACACGATTCCTAGTGGAACATACAACGATGACCCATTCACTACCTCTATTCATATTCCAACACTTTATGGTTCAAACTTCGGGAGGGGCAAAATCATTGTATTGGAGCCTGATGGTGAGATAACCGTGTTTGAGCAACCTACGGCTGGATTGAATATCGAacc                                                                                                                                                                                                                                                              >  1:180358/1‑301 (MQ=255)`  
                                                  `gTATCAGCTCAGATATGTATGTGATTCTTCGCAATGAGTTCAATACGTTCCTAGCTAGAATCTCTTTCACTAAGAACGCCATTGACTTACAGGGAGAACCCTATCGTGCCTTTATGGACATGAAGATTCGATACACGATTCCTAGTGGAACATACAACGATGACACATTCACTACCTCTATTCATATTCCAACAATTTATGGTGCAAACTTCGGGAGGGGCAAAATCACTGTATTGGAGCCTGATGGTGAGATAACCGTGTTTGAGCAACCTACGGCTGGGTGGAATAGCGACCCTTGGCt                                                                                                                                                                                                                                                        <  1:52690/301‑1 (MQ=255)`  
                                                   `tATCAGCTCAGATATGTATGTGATTCTTCGCAATGAGTTCAATACGTTCCTAGCTAGAATCTCTTTCACTAAGAACGCCATTGACTTACAGGGAGAACCCTATCGTGCCTTTATGGACATGAAGATTCGATACACGATTCCTAGTGGAACATACAACGATGACACATTCACTACCTCTATTCATATTCCAACAATTTATGGTGCAAACTTCGGGAGGGGCAAAATCACTGTATTGGCGCCTGATGGTGAGATAACCGTGTTTGAGCAACCTACGGCTGGGTGGAATAGCGACCCTTGGCTg                                                                                                                                                                                                                                                       >  1:127751/1‑301 (MQ=255)`  
                                                   `tATCAGCTCAGATATGTATGTGATTCTTCGCAATGAGTTCAATACGTTCCTAGCTAGAATCTCTTTCACTAAGAACGCCATTGACTTACAGGGAGAACCCTATCGTGCCTTTATGGACATGAAGATTCGATACACGATTCCTAGTGGAACATACAACGATGACACATTCACTACCTCTATTCATATTCCAACAATTTATGGTGCAAACTTCGGGAGGGGCAAAATCACTGTATTGGAGCCTGATGTTGAGATAACCGTGTTTGAGCAACCTACGGCTGGGTGGAATAGCGACCCTTTGCTg                                                                                                                                                                                                                                                       >  1:51906/1‑301 (MQ=255)`  
                                                       `ataagagacaggtatgtGATTCTTCGCAATGAGTTCAATACGTTCCTAGCTAGAATCTCTTTCACTAAGAACGCCATTGACTTACAGGGAGAACCCTATCGTGCCTTTATGGACATGAAGATTCGATACACGATTCCTAGTGGAACATACAACGATGACACATTCACTACCTCTATTCATATTCCAACAATTTATGGTGCAAACTTCGGGAGGGGCAAAATCACTGTATTGGAGCCTGATGGTAAGATAACCGTGTTTGAGCAACCTACGGCTGGGTGGAATAGCGACCCTTGGCTGAGAc                                                                                                                                                                                                                                                   <  1:586602/290‑1 (MQ=255)`  
                                                            `aGATATGTATGTGATTCTTCGCAATGAGTTCAATACGTTCCTAGCTAGAATCTCTTTCACTAAGAACGCCATTGACTTACAGGGAGAACCCTATCGTGCCTTTATGGACATGAAGATTCGATACACGATTCCTAGTGGAACATACAACGATGACACATTCACTACCTCTATTCATATTCCAACAATTTATGGTGCAAACTTCGGGAGGGGCAAAATAACTGTATTGGAGCCTGATGGTGAGATAACCGTGTTTGAGCAACCTACGGCTGGGTGGAATAGCGACCCTTGGCTGAGACTCAGc                                                                                                                                                                                                                                              >  1:531409/1‑301 (MQ=255)`  
                                                             `gATATGTATGTGATTCTTCGCAATGAGTTCAATACGTTCCTAGCTAGAATCTCTTTCACTAAGAACGCCATTGACTTACAGGGAGAACCCTATCGTGCCTTTATGGACATGAAGATTCGATACACGATTCCTAGTGGAACATACAACGATGACACATTCACTACCTCTATTCATATTCCAACAATTTATGGTGCAAACTTCGGGAGGGGCAAAATCACTGTATTGGAGCCTGATGGTGAGCTAACCGTGTTTGAGCAACCTACGGCTGGGTGGAATAGCGACCCTTGGCTGAGACTCATCg                                                                                                                                                                                                                                             >  1:506223/1‑301 (MQ=255)`  
                                                             `gATATGTATGTGATTCTTCGCAATGAGTTCAATACGTTCCTAGCTAGAATCTCTTTCACTAAGAACGCCATTGACTTACAGGGAGAACCCTATCGTGCCTTTATGGACATGAAGATTCGATACACGATTCCTAGTGGAACATACAACGATGACACATTCACTACCTCTATTCATATTCCAACAATTTATGGTGCAAACTTCGGGAGGGGCAAAATCACTGTATTGGAGCCTGATGGTGAGATAACCGTGTTTGAGCAACCTACGGCTGGGTGGAATAGCGACCCTTGGCTGAGACTCAGCg                                                                                                                                                                                                                                             >  1:345708/1‑301 (MQ=255)`  
                                                             `gATATGTATGTGATTCTTCGCAATGAGTTCAATACGTTCCTAGCTAGAATCTCTTTCACTAAGAAAGCCATTGACTTACAGGGAGAACCCTATCGTGCCTTTATGGACATGAAGATTCGATACACGATTCCTAGTGGAACATACAACGATGACACATTCACTACCTCTATTCATATTACAACAATTTATGGTGCAAACTTCGGGAGGGGCAAAATCACTGGATTGGCGCCTGATGGTGAGATAACCGTGTTTGAGCAACCTACGGCTGGGTGGAATAGCGACCCTTGGCTGAGACTCAGCg                                                                                                                                                                                                                                             >  1:201021/1‑301 (MQ=255)`  
                                                              `gtataagagacagtcttcGCAATGAGTTCAATACGTTCCTAGCTAGAATCTCTTTCACTAAGAACGCCATTGACTTACAGGGAGAACCCTATCGTGCCTTTATGGACATGAAGATTCGATACACGATTCCTAGTGGAACATACAACGATGACACATTCACTACCTCTATTCATATTCCAACAATTTATGGTGCAAACTTCGGGAGGGGCAAAATCACTGTATTGGAGCCTGATGGTGAGATAACCGTGTTTGAGCAACCTACGGCTGGGTGGAATAGCGACCCTTGGCTGAGACTCAGCgg                                                                                                                                                                                                                                            <  1:188222/288‑1 (MQ=255)`  
                                                                `atgtatgtGATTCTTCGCAATGAGTTCAATACGTTCCTAGCTAGAATCTCTTTCACTAAGAACGCCATTGACTTACAGGGAGAACCCTATCGTGCCTTTATGGACATGAAGATTCGATACACGATTCCTAGTGGAACATACAACGATGACACATTCACTACCTCTATTCATATTCCAACAATTTATGGTGCAAACTTCGGGAGGGGCAAAATCACTGTATTGGAGCCTGATGGTGAGATAACCGTGTTTGAGCAACCTACGGCTGGGTGGAATAGCGACCCTTGGCTGAGACTCAGCGGTa                                                                                                                                                                                                                                          >  1:305015/1‑301 (MQ=255)`  
                                                                 `tgtatgtGATTCTTCGCAATGAGTTCAATACGTTCCTAGCTAGAATCTCTTTCACTAAGAACGCCATTGACTTACAGGGAGAACCCTATCGTGCCTTTATGGACATGAAGATTCGATACACGATTCCTAGTGGAACATACAACGATGACACATTCACTACCTCTATTCATATTCCAACAATTTATGGTGCAAACTTCGGGAGGGGCAAAATCACTGTATTGGAGCCTGATGGTGAGATAACCGTGTTTGAGCAACCTACGGATGGGTGGAATAGCGACCCTTGGCTGAGACTCAGCGGTaa                                                                                                                                                                                                                                         >  1:217465/1‑301 (MQ=255)`  
                                                                  `gtatgtGATTCTTCGCAATGAGTTCAATACGTTCCTAGCTAGAATCTCTTTCACTAAGAACGCCATTGACTTACAGGGAGAACCCTATCGTGCCTTTATGGACATGAAGATTCGATACACGATTCCTAGTGGAACATACAACGATGACACATTCACTACCTCTATTCATATTCCAACAATTTATGGTGCAAACTTCGGGAGGGGCAAAATCACTGTATTGGAGCCTGATGGTGAGCTAACCGTGTTTGAGCAACCTACGGCTGGGTGGAATAGCGACCCTTGGCTGAGACTCCGCGGTAAc                                                                                                                                                                                                                                        >  1:561497/1‑301 (MQ=255)`  
                                                                  `gtatgtGATTCTTCGCAATGAGTTCAATACGTTCCTAGCTAGAATCTCTTTCACTAAGAACGCCATTGACTTACAGGGAGAACCCTATCGTGCCTTTATGGACATGAAGATTCGATACACGATTCCTAGTGGAACATACAACGATGACACATTCACTACCTCTATTCATATTCCAACAATTTATGGTGCAAACTTCGGGAGGGGCAAAATCACTGTATTGGAGCCTGATGGTGAGATAACCGTGTTTGAGCAACCTACGGCTGGGTGGAATAGCGACCCTTGGCTGAGACTCAGCGGTAAc                                                                                                                                                                                                                                        >  1:254754/1‑301 (MQ=255)`  
                                                                  `gtatgtGATTCTTCGCAATGAGTTCAATACGTTCCTAGCTAGAATCTCTTTCACTAAGAACGCCATTGACTTACAGGGAGAACCCTATCGTGCCTTTATGGACATGAAGATTCGATACACGATTCCTAGTGGAACATACAACGATGAAACATTCACTACCTCTATTCATATTCCAACAATTTATGGTGCAAACTTCGGGAGGGGCAAAATCACTGTATTGGAGCCTGATGGTGAGATAACCGTGTTTGAGCAACCTACGGCTGGGTGGAATAGCGACCCTTGGCTGAGACTCAGCGGTAAc                                                                                                                                                                                                                                        >  1:36925/1‑301 (MQ=255)`  
                                                                  `gtatgtGATTCTTCGCAATGAGTTCAATACGTTCCTAGCTAGAATCTCTTTCACTAAGAACGCCATTGACTTACAGGGAGAACCCTATCGTGCATTTATGGACATGAAGATTCGATACACGATTCCTAGTGGAACATACAACGATGACACATTCACTACCTCTATTCATATTCCAATAATTTATGGTGCAAACTTCGGGCGGGGCAAAATCACTGTATTGGAGCCTGATGGTGAGATAACCGTGTTTGAGCAACCTACGGCTGGGTGGAATAGCGACCCTTGGCTGAGATTCACTGGTAAc                                                                                                                                                                                                                                        >  1:79707/1‑301 (MQ=255)`  
                                                                    `ttgtgATTCTTCGCAATGAGTTCAATACGTTCCTAGATAGAATCTCTTTCACTAAGAACGCCATTGACTTACAGGGAGAAACCTATCGTGCCTTTATGGACATGAAGATTCGATACACGATTCCTAGTGGAACATACAACGATGACACATTCACTACCTCTATTCATATTCCAACAATTTATGGTGCAAACTTCGGGAGGGGCAAAATCACTGTATTGGAGCCTGATGGTGAGATAACCGTGTTTGAGCAACCTACGGCTGGGTGGAATAGCGACCCTTGGCTGAGACTCAGCGGTAACtt                                                                                                                                                                                                                                      <  1:380203/300‑1 (MQ=255)`  
                                                                         `aTTCTTCGCAATGAGTTCAATACGTTCCTAGCTAGAATCTCTTTCACTAAGAACGCCATTGACTTACAGGGAGAACCCTATCGTGCCTTTATGGACATGAAGATTCGATACACGATTCCTAGTGGAACATACAACGATGACACATTCACTACCTCTATTAATATTCCAACAATTTATGGTGCAAACTTCGGGAGGGGCAAAGTCACTGTATTGTCGCCTGATGGTGAGATAACCGTGTGTGAGCAACCTACGGCTGGGTGGAATAGCGACCCTTGTCTGAGACTCATCGGTAACTtggtgg                                                                                                                                                                                                                                 >  1:636439/1‑301 (MQ=255)`  
                                                                              `tcGCAATGAGTTCAATACGTTCCTAGCTAGAATCTCTTTCACTAAGAACGCCATTTACTTACAGGGAGAACCCTATCGTGCCTTTATGGACATGAAGATTCGATACACGATTCCTAGTGGAACATACAACGATGACACATTCACTACCTCTATTCATATTCCAACAATTTATGGTGCAAACTTCGGGAGGGGCAAAATCACTGTGTTGGAGCCTGATGGTGAGATAACCGTGTTTGAGCAACCTACGGCTGGGTGGAATAGCGACCCTTGGCTGAGACTAAGCGGTAACTTggctgtctct                                                                                                                                                                                                                            >  1:653660/1‑293 (MQ=255)`  
                                                                                     `gAGTTCAATACGTTCCTAGCTAGAATCTCTTTCACTAAGAACGCCATTGACTTACAGGGAGAACCCTGTCGTGCCTTTATGGACATGAAGATTCGATACACGATTCCTAGTGGAACATACAACGATGACACATTCACTACCTCTATTCATATTCCAACAATTTATGGTGCAAACTTCGGGAGGGGCAAAATCACTGTATTGGAGCCTGATGGTGAGATAACCGTGTTTGAGCAACCTACGGCTGGGTGGAATAGCGACCCTTGGCTGAGACTCAGCGGTAACTTGGGGGGACGCATGgtgt                                                                                                                                                                                                                     >  1:475440/1‑301 (MQ=255)`  
                                                                                       `gTTCAATACGTTCCTAGCTAGAATCTCTTTCACTAAGAACGCCATTGACTTACAGGGAGAACCCTATCGTGCCTTTATGGACATGAAGATTCGATACACGATTCCTAGTGGAACATACAACGATGACACATTCACTACCTCTATTCATATTCCAACAATTTATGGTGCAAACTTCGGGAGGGGCAAAATCACTGTATTGGAGCCTGATGGTGAGATAACCGTGTTTGAGCAACCTACGGCTGGGTGGAATAGCGACCCTTGGCTGAGACTCAGCGGTAACTTGGAGGGACGCATGGTGTAc                                                                                                                                                                                                                   >  1:23681/1‑301 (MQ=255)`  
                                                                                         `tgaaTACGTTCCTAGCTAGAATCGCTTTCACTAAGAACGCCATTGACTTAAAGGGAGAAACCTATCGTGCCTTTATGGACATGAAGATTCGATAAACGATGCCTAGTGGAACAGACAACGAAGACACATTCACTACCTCTATTCATATTCCAACAAGTTATGGTGCAAACTTCGGGAGGGGCAAAATCACTGTATTGGAGCCTGATGGTGAGATAACCGTGTTTGAGCAACCTACGGCTGGGTGGAATAGCGACCCTTGGCTGAGACTCAGCGGTAACTTGGAGGGACGCATGGTGTACAt                                                                                                                                                                                                                 <  1:386304/299‑1 (MQ=255)`  
                                                                                         `tCAATACGTTCCTAGCTAGAATCTCTTTCACTAAGAACGCCATTGACTTACAGGGAGAACCCTATCGTGCCTTTATGGACATGAAGATTCGATACACGATTCCTAGTGGAACATACAACGATGACACATTCACTACCTCTATTCATATTCCAACAATTTATGGTGCAAACTTCGGGAGGGGCAAAATCACTGTATTGGAGCCCGATGGTGAGATAACCGTGTTTGAGCAACCTACGGCTGGGTGGAATAGCGACCCTTGGCTGAGACTCAGCGGTAACTTGGAGGGACGCATGGTGTACAt                                                                                                                                                                                                                 <  1:260648/301‑1 (MQ=255)`  
                                                                                         `tCAATACGTTCCTAGCTAGAATCTCTTTCACTAAGAACGCCATTGACTTACAGGGAGAACCCTATCGTGCCTTTATGGACAGGAAGATTCGATACACGATTCCTAGTGGAACATACAACGATGACACATTCACTACCTCTATTCATATTCCAACAATTTATGGTGCAAACTTCGGGAGGGGCAAAATCACGGTATTGGAGCCTGATGGTGAGATAACCGTGTTTGAGCAACCTACGGCTGGGTGGAATAGCGACCCTTGGCTGAGACTCAGCGGTAACTTGGAGGGACGCATGGTGTACAt                                                                                                                                                                                                                 <  1:84903/301‑1 (MQ=255)`  
                                                                                         `tCAATACGTTACTAGCTAGAATCTCTTTCACTAAGAACGCCATTGACTTACAGGGAGAACCCTATCGTGCCTTTATGGACATGAAGATTCGATACACGATTCCTAGTGGAACATACAACGATGACACATTCACTACCTCTATTCATATTCCAACAATTTATGGTGCAAACTTCGGGAGGGGCAAAATCACTGTATTGGAGCCTGATGGTGAGATAACCGTGTTTGAGCAACCTACGGCTGGGTGGAATAGCGACCCTTGGCTGAGACTCAGCGGTAACTTGGAGGGACGCATGGTGTACAt                                                                                                                                                                                                                 <  1:628483/301‑1 (MQ=255)`  
                                                                                          `tgtataagagacagctagAATCTCTTTCACTAAGAACGCCATTGACTTACAGGGAGAACCCTATCGTGCCTTTATGGACATGAAGATTCGATACACGATTCCTAGTGGAACATACAACGATGACACATTCACTACCTCTATTCATATTCCAACAATTTATGGTGCAAACTTCGGGAGGGGCAAAATCACTGTATTGGAGCCTGATGGTGAGATAACCGTGTTTGAGCAACCTACGGCTGGGTGGAATAGCGACCCTTGGCTGAGACTCAGCGGTAACTTGGAGGGACGCATGGTGTACAtt                                                                                                                                                                                                                <  1:574040/289‑1 (MQ=255)`  
                                                                                          `cAATACGTTCCTAGCTAGAATCTCTTTCACTAAGAACGCCATTGACTTACAGGGAGAACCCTATCGTGCCTTTATGGACATGAAGATTCGATACACGATTCCTAGTGGAACATACAACGATGACACATTCACTACCTCTATTCATATTCCAACAATTTATGGTGCAAACTTCGGGAGGGGCAAAATCACTGTATTGGAGCCTGATGGTGAGATAACCGTGTTTGAGCAACCTACGGCTGGGTGGAATAGCGACCCTTGGCTGCGACTCAGCGGTAACTTGGAGGGACGCATGGTGTACAtt                                                                                                                                                                                                                >  1:597547/1‑301 (MQ=255)`  
                                                                                             `tACGTTCCTAGCTAGAATCTCTTTCACTAAGAACGCCATTGACTTACAGGGAGAACCCTATCGTGCCTTTATGGACATGAAGATTCGATACACGATTCCTAGTGGAACATACAACGATGACACATTCACTACCTCTATTCATATTCCAACAATTTATGGTGCAAACTTCGGGAGGGGCAAAATCACTGTATTGGAGCCTGATGGTGAGATAACCGTGTTTGAGCAACCTACGGCTGGGTGGAATAGCGACCCTTGGCTGAGACTCAGCGGTAACTTGGAGGGACGCATGGTGTACATTggg                                                                                                                                                                                                             <  1:298014/301‑1 (MQ=255)`  
                                                                                              `aCGTTCCTAGCTAGAATCTCTTTAACTAAGAACGCCATTGACTTACAGGGAGAACCCTATCGTGCCTTTATGGACATGAAGATTCGATACACGATTCCTAGTGGAACATACAACGATGACACATTCATTACCTCTATTCATATTCAAACAATTTATGGTGCAAACTTCGGGAGGGGCAAAATCACTGTATTGGAGCCTGATGGTGAGATAACCGTGTTTGAGCAACCTACGGCTGGGTGGAATAGCGACCCTTGGCTGAGACTCAGCGGTAACTTGGAGGGACGCATGGTGTACATTGGGt                                                                                                                                                                                                            <  1:139495/301‑1 (MQ=255)`  
                                                                                                `tttCCTAGCTAGAATCTCTTTCACTAAGAACGCCATTGACTTACAGGGAGAACCCTATCGTGCATTTATGGACATGAAGATTCGATACACGATTCCTAGTGGAACATACAACGATGACACATTCACTACCTCTATTCATATTCCAACAATTTATGGTGCAAACTTCGGGAGGGGCAAAATCACTGTATTGGAGCCTGATGGTGAGATAACCGTGTTTGAGCAACCTACGGCTGGGTGGAATAGCGACCCTTGGCTGAGACTCAGCGGTAACTTGGAGGGACGCATGGTGTACCTTCGTTTc                                                                                                                                                                                                          <  1:610174/300‑1 (MQ=255)`  
                                                                                                 `ttCCTAGCTAGAATCTCTTTCACTAAGAACGCCATTGACTTACAGGGAGAACCCTATCGTGCCTTTATGGACATGAAGATTCGATACACGATTCCTAGTGGAACATACAACGATGACACATTCACTACCTCTATTCATATTCCAACAATTTATGGTGCAAACTTCGGGAGGGGCAAAATCACTGTATTGGAGACTGATGGTGAGATAACCGTGTTTGAGCAACCTACGGCTGGGTGGAATAGCGACCCTTGGCTGAGACTCAGCGGTAACTTGGAGGGACGCATGGTGTACATTGGGTTCa                                                                                                                                                                                                         >  1:455135/1‑301 (MQ=255)`  
                                                                                                       `gctagAATCTCTTTCACTAAGAACGCCATTGACTTACAGGGAGAACCCTATCGTGCCTTTATGGACATGAAGATTCGATACACGATTCCTAGTGGAACATACAACGATGACACATTCACTACCTCTATTCATATTCCAACAATTTATGGTGCAAACTTCGGGAGGGGCAAAATCACTGTATTGGAGCCTGATGGTGAGATAACCGTGTTTGAGCAACCTACGGCTGGGTGGAATAGCGACCCTTAGCTGAGACTCAGCGGTAACTTGGAGGGACGCATGGTGTACATTGGGTTCAACATTa                                                                                                                                                                                                   >  1:195730/1‑301 (MQ=255)`  
                                                                                                          `gggctcggagatgtgtataagagacaggACTTACAGGGAGAACCCTATCGTGCCTTTATGGACATGAAGATTCGATACACGATTCCTAGTGGAACATACAACGATGACACATTCACTACCTCTATTCATATTCCAACAATTTATGGTGCAAACTTCGGGAGGGGCAAAATCACTGTATTGGAGCCTGATGGTGAGATAACCGTGTTTGAGCAACCTACGGCTGGGTGGAATAGCGACCCTTGGCTGAGACTCAGCGGTAACTTGGAGGGACGCATGGTGTACATTGGGTTCAACATTAACt                                                                                                                                                                                                <  1:94478/274‑1 (MQ=255)`  
                                                                                                                   `ttCACTAAGAACGCCATTGACTTACAGGGAGAACCCTATCGTGCCTTTATGGACATGAAGATTCGATACACGATTCCTAGTGGAACATACAACGATGACACATTCACTACCTCTATTCATATTCCAACAATTTATGGTGCAAACTTCGGGAGGGGCAAAATCACTGTATTGGAGCCTGATGGTGAGATAACCGTGTTTGAGCAACATACGGCTGGGTGGAATAGCGACCCTTGGCTGAGACTCAGCGGTAACTTGGAGGGACGCATGGTGTACATTGGGTTCAACATTAACTTCGTATATg                                                                                                                                                                                       >  1:338752/1‑301 (MQ=255)`  
                                                                                                                     `tgactaGAACTCCCTTTACCTACAGGGAGCACCCTATCGTGCCTTTCTGGACATGAATATTCGATACACGATTCCTAGTGGAACATACATCGCTGACACATTCACTACCTCTATTCATATTCCAACAATTTATGGTGCAAACTTCGGGTGGGGCAAAATCACTGGATTGGAGCCTGATGGTGAGCTCTCCGTGTTTAAGCAACCTACGGCTGGGTAGAATAGCGACCCTTGGCTGAGACTCAGCGGTAACTTGGAGGGACGCATGGTGTACATTGGGTTCAACATTAACTTCATATATGAg                                                                                                                                                                                     <  1:100126/296‑1 (MQ=255)`  
                                                                                                                       `cTAAGAACGCCATTGACTTACAGGGAGAACCCTATCGTGCCTTTATGGACATGAAGATTCGATACACGATTCCTAGTGGAACATACAACGATGACACATTCACTACCTCTATTCATATTCCAACAATTTATGGTGCAAACTTCGGGAGGGGCAAAATCACTGTATTGGAGCCTGATGGTGAGATAACCGTGTTTGAGCAACCTACGGCTGGGTGGAATAGCGACCCTTGGCTGAGACTCAGCGGTAACTTGGAGGGACGCATGGTGTACATTGGGTTCAACATTAACTTCGTATATGAGtt                                                                                                                                                                                   >  1:272303/1‑301 (MQ=255)`  
                                                                                                                         `aaGAACGCCATTGACTTACAGGGAGAACCCTATCGTGCCTTTATGGACATGAAGATTCGATACACGATTCCTAGTGGAACATACAACGATGACACATTCACTACCTCTATTCATATTCCAACAATTTATGGTGCAAACTTCGGGAGGGGCAAAATCACTGTATTGGAGCCTGATGGTGAGATAACCGTGTTTGAGCAACCTACGGCTGGGTGGAATAGCGACCCTTGGCTGAGACTCAGCGGTAACTTGGAGGGACGCATGGTGTACATTGGGTTCAACATTAACTTCGTATATGAGTtct                                                                                                                                                                                 >  1:319176/1‑301 (MQ=255)`  
                                                                                                                         `aaGAACGCCATTGACTTACAGGGAGAACCCTATCGTGCCTTTATGGACATGAAGATTCGATACACGATTCCTAGTGGAACATACAACGATGACACATTCACTACCTCTATTCATATTCCAACAATTTATGGTGCAAACTTCGGGAGGGGCAAAATCACTGTATTGGAGCCTGATGGTGAGATAACCGTGTTTGAGCAACCTACGGCTGGGTGGAATAGCGACCCTTGGCTGAGACTCAGCGGTAACTTGGAGGGACGCATGGTGTACATTGGGTTCAACATTAACTTCGTATATGAGTtct                                                                                                                                                                                 >  1:231146/1‑301 (MQ=255)`  
                                                                                                                                 `aagagacagaCAGGGAGAACCATATCGTGCCTTTATGGACATGAAGATTCGATACACGATTCCTAGTGGAACATACAACGATGACACATTCACTACCTCTATTCATATTCCAACAATTTATGGTGCAAACTTCGGGAGGGGCAAAATCACTGTATTGGAGCCTGATGGTGAGATAACCGTGTTTGAGCAACCTACGGCTGGGTGGAATAGCGACCCTTGGCTGAGACTCAGCGGTAACTTGGAGGGACGCATGGTGTACATTGGGTTCAACATTAACTTCGTATATGAGTTCTCTAAGTTc                                                                                                                                                                         <  1:358527/292‑1 (MQ=255)`  
                                                                                                                                             `gggAGAACCCTATCGTGCCTTTATGGACATGAAGATTCGATACACGATTCCTAGTGGAACATACAACGATGACACATTCACTACCTCTATTCATATTCCAACAATTTATGGTGCAAACTTCGGGAGGGGCAAAATCACTGTATTGGAGCCTGATGGTGAGATAACCGTGTTTGAGCAACCTACGACTGGGTGGAATAGCGACCCTTGGCTGAGACTCAGCGGTAACTTGGAGGGACGCATGGTGTACATTGGGTTCAACATTAACTTCGTATATGAGTTCTCTAAGtctgtctcttataca                                                                                                                                                             >  1:28329/1‑287 (MQ=255)`  
                                                                                                                                               `aagagacagaTCGTGCCTTTATGGACATGAAGATTCGATACGCGATTCCTAGTGGAACATACAACGATGACACATTCACTACCTCTATTCATATTCCAACAATTTATGGTGCAAACTTCGGGAGGGGCAAAATCACTGTATTGGAGCCTGATGGTGAGATAACCGTGTTTGAGCAACCTACGGCTGGGTGGAATAGCGACCCTTGGCTGAGACTCAGCGGTAACTTGGAGGGACGCATGGTGTACATTGGGTTCAACATTAACTTCGTATATGAGTTCTCTAAGTTCCTCATCAAGCAGAc                                                                                                                                                           <  1:538259/292‑1 (MQ=255)`  
                                                                                                                                                `agaACCCTATCGTGCCTTTATGGACATGAAGATTCGATACACGATTCCTAGTGGAACATACAACGATGACACATTCACTACCTCTATTCATATTCCAACAATTTATGGTGCACACTTCGGGAGGGGCAAAATCACTGTATTGGAGCCTGATGGTGCGATAACCGTGTTTGAGCAACCTACGGCTGGGTGGAATAGCGACCCTTGGCTGAGACTCAGCGGTAACTTGGAGGGACGCATGGTGTACATTGGGTTCAACATTAACTTCGTATATGAGTTCTCTAAGTTCCTCATCAAGCAGACt                                                                                                                                                          <  1:31210/301‑1 (MQ=255)`  
                                                                                                                                                  `gagatgtgtataagagacaggACATGAAGATTCGATACACGATTCCTAGTGGAACATACAACGATGACACATTCACTACCTCTATTCATATTCCAACAATTTATGGTTCAAACTTCGGGAGGGGCAAAATCACTGTATTGGAGCCTGATGGTGAGATAACCGTGTTTGAGCAACCTACGGCTGGGTGGAATAGCGACCCTTGGCTGAGACTCAGCGGTAACTTGGAGGGACGCATGGTGTACATTGGGTTCAACATTAACTTCGTATATGAGTTCTCTAAGTTCCTCATCAAGCAGACTGc                                                                                                                                                        <  1:148865/282‑1 (MQ=255)`  
                                                                                                                                                   `aCCCTATCGTGCCTTTATGGACATGAAGATTCGATACACGATTCCTAGTGGAACATACAACGATGACACATTCACTACCTCTATTCATATTCCAACAATTTATGGTGCAAACTTCGGGAGGGGCAAAATCACTGTATTGGAGCCTGATGGTGAGATAACCGTGTTTGAGCAACCTACGGCTGGGTGGAATAGCGACCCTTGGATGAGACTCAGCGGTAACTTGGAGGGACGCATGGTGTACATTGGGTTCAACATTAACGTCGTATATGAGTTGTCTAAGTTCCTCATCAAGAAGACTGcc                                                                                                                                                       >  1:89384/1‑301 (MQ=255)`  
                                                                                                                                                   `aCCCTATCGTGCCTTTATGGACATGAAGATTCGATACACGATTCCTAGTGGAACATACAACGATGACACATTCACTACCTCTATTCATATTCCAACAATTTATGGTCCAAACTTCGGGAGGGGCAAAATCACTGTATTTGAGCCTGATGGTGAGATAACCGTGTTTGAGCAACTTACGGCT‑GGTGGAATAGCGACCCTTGGCTGTGACTCAGCGGTAACTTGGAGGGACGCATGGTGTACATTGGGTTCAACATTCACTTCGTATATGAGTTCTCTAAGTTCCtcatctgtctcttattta                                                                                                                                                      >  1:398037/1‑288 (MQ=255)`  
                                                                                                                                                        `aTCGTGCCTTTATGGACATGAAGATTCGATACACGATTCCTAGTGGAACATACAACGATGACACATTCACTACCTCTATTCATATTCCAACAATTTATGGTGCAAACTTCGGGAGGGGCAAAATCACTGTATTGGAGCCTGATGGTAAGATAACCGTGTTTGAGCAACCTACGGCTGGGTGGAATAGCGACCCTTGGCTGAGACTCAGCGGTAACTTGGAGGGACGCAGGGTGTACATTGGGTTCAACATTAACTTCGTATATGAGTTCTCTAAGTTCCTCATCAAGCAGACTGCcgacga                                                                                                                                                  >  1:162809/1‑301 (MQ=255)`  
                                                                                                                                                              `ccTTTATGGACATGAAGATTCGATACACGATTCCTAGTGGAACATACAACGATGACACATTCACTACCTCTATTCATATTCCAACAATTTATGGTGCAAACTTCGGGAGGGGCAAAATCACTGTATTGGAGCCTGATGGTGAGATAACCGTGTTTGAGCAACCTACGGCTGGGTGGAATAGCGACCCTTGGCTGAGACTCAGCGGTAACTTGGAGGGACGCATGGTGTACATTGGGTTCAACATTAACTTCGTATATGAGTTCTCTAAGTTCCTCATCAAGCAGACTGCCGACGACGGGTc                                                                                                                                            >  1:421381/1‑301 (MQ=255)`  
                                                                                                                                                                 `ttATGGACATGAAGATTCGATACACGATTCCTAATGGAACATACAACGATGACACCTTCACTACCTCTATTCATATTCCAACAATTTATTGTGCAAACTTCGGGAGGGGCAAAAGCACTGTATTGGAGCCTGATGGTGAGATAACCGTGTTTGAGCAACCTACGGCTGGGTGGAATAGCGACCCTTGGCTGAGACTCAGCGGTAACTTGGAGGGACGCATGGTGTACATTGGGTTCAACATTAACTTCGTATATGAGTTCTCTAAGTTCCTCATCAAGCAGACTGCCGACGACGGGTCTAc                                                                                                                                         <  1:293710/301‑1 (MQ=255)`  
                                                                                                                                                                    `aggACATGAAGATTCGATACACGATTCCTAGTGGAACATACAACGATTACACATTCACTACCTCTATTCATATTCCAACAATTTATGGTGCAAACTTCGGGAGGGGCAAAATCACTGTATTGGAGCCTGATGGTGAGATAACCGTGTTTGAGCAACCTACGGCTGGGTGGAATAGCGACCCTTGGCTGAGACTCAGCGGTAACTTGGAGGGACGCATGGTGTACATTGGGTTCAACATTAACTTCGTATATGAGTTCTCTAAGTTCCTCATCAAGCAGACTGCCGACGACGGGTCTACCTc                                                                                                                                      <  1:415447/300‑1 (MQ=255)`  
                                                                                                                                                                     `cggagatgggtataagagacagATTCCTAGTGGAACATACAACGATGACACATTCACTACCTCTATTCATATTCCAACAAATTATGGTGCAAACTTCGGGAGGGGCAAAATCACTGTATTGGAGCCTGATGGTGAGATAACCGTGTTTGAGCAACCTACGGCTGGGTGGAATAGCGACCCTTGGCTGAGACTCAGCGGTAACTTGGAGGGACGCATGGTGTACATTGGGTTCAACATTAACTTCGTATATGAGTTCTCTAAGTTCCTCATCAAGCAGACTGCCGACGACGGGTCTACCTcc                                                                                                                                     <  1:462056/280‑1 (MQ=255)`  
                                                                                                                                                                      `gACATGAAGATTCGATACACGATTCCTAGTGGAACATACAACGATGACACATTCACTACCTCTATTCATATTCCAACAATTTATGGTGCAAACTTCGGGAGGGGCAAAATCACTGTATTGGAGCCTGATGGTGAGATAACCGTGTTTGAGCAACCTACGGCTGGGTGGAATAGCGACCCTTGGCTGAGACTCAGCGGTAACTTGGAGGGACGCATGGTGTACATTGGGTTCAACATTAACTTCGTATATGAGTTCTCTAAGTTCCTCATCAAGCAGACTGCCGACGACGGGTcctgtccct                                                                                                                                    >  1:331999/1‑293 (MQ=255)`  
                                                                                                                                                                         `aTGAAGATTCGATACACGATTCCTAGTGGAACATACAACGATTACACATTCACTACCTCTATTCATATTCCAACAATTTATGGTGCAAACTTCGGGAGGGGCAAAATCACTGTATTGGAGCCTGATGGTGAGATAACCGTGTTTGAGCAACCTACGGCTGGGTGGAATAGCGACCCTTGGCTGAGACTCAGCGGTAACTTGGAGGGACGCATGGTGTACATTGGGTTCAACATTAACTTCGTATATGAGTTCTCTAAGTTCCTCATCAAGCAGACTGCCGACGACGGGTCTACCTCCACgg                                                                                                                                 <  1:67529/301‑1 (MQ=255)`  
                                                                                                                                                                            `ataagagacagcacGATTCCTAGTGGAACATACAACGATGACACATTCACTACCTCTATTCATATTCCAACAATTTATGGTGCAAACTTCGGGAGGGGCAAAATCACTGTATTGGAGACTGATGGTGAGATAACCGTGTTTGAGCAACCTACGGCTGGGTGGAATAGCGACCCTTGGCTGAGACTCAGCGGTAACTTGGAGGGACGCATGGTGTACATTGGGTTCAACATTAACTTCGTATATGAGTTCTCTAAGTTCCTCATCAAGCAGACTGCCGACGACGGGTCTACCTCCACGGAAg                                                                                                                              <  1:448049/290‑1 (MQ=255)`  
                                                                                                                                                                                         `cGATTCCTAGTGGAACATACAACGGTGACACATTCACTACCCCTATTCATATTCCAACAATTTATGGTGCAAACTTCGGGAGGGGCAAAATCACTGTATTGGAGCCTGATGGTGAGATAACCGTGTTTGAGCAACCTACGGCTGGGTGGAATAGCGACCCTTGGCTGAGACTCAGCGGTAACTTGGAGGGACGCATGGTGTACATTGGGTTCAACATTAACTTCGTATATGAGTTCTCTAAGTTCCTCATCAAGCAGACTGCCGGCGACGGGTCTAccctgtctcttatacacatctccga                                                                                                                 >  1:169279/1‑278 (MQ=255)`  
                                                                                                                                                                                           `aTTCCTAGTGGAACATACAACGATGACACATTCACTACCTCTATTCATATTCCAACAATTTATGGTGCAAACTTCTGGAGGGGCAAAATCACTGTATTGGAGCCTGATGGTGAGATAACCGTGTTTGAGCAACCTACGGCTGGGTGGAATAGCGACACTTGGCTGAGACTCAGCGGTAACTTGGAGGGACGCATGGTGTACATTGGGTTCAACATTAACTTCGTATATGAGTACTCTAAGTTCCTCATCAAGCAGACTGCCGACGATGGGTCTACCTCCACGGAAGACATTGGGCGCTTAc                                                                                                               >  1:404014/1‑301 (MQ=255)`  
                                                                                                                                                                                                   `gcgAACATACAACGATGACACATTCACTACCTCTATTCCTATCCCAACAATTTATGGTGCAAACTTCGGGAGGGGCAAAATCACTGTATTGGAGCCTGATGGTGAGATAACCGTGTTTGAGCAACCTACTGCTGGGTTGAATAGCGACCCTTGGCTGAGACGCAGCGGTAACTTGGAGGGACGCATTGTGTATATTGGGTTCAACATTAACTTCGTATATGAGTTCTCTAAGTTCCTCATCAAGCAGACTGCCGACGACGGGTCTACCTCCACGGAAGACATTGGGCGCTTACAGTTACGc                                                                                                       <  1:267043/299‑1 (MQ=255)`  
                                                                                                                                                                                                    `ggAACATACAACGATGACACATTCACTACCTCTATTCATATTCCAACAATTTATGGTGCAAACTTCGGGAGGGGCAAAATCACTGTATTGGAGCCTGATGGTGAGATAACCGTGTTTGAGCAACCTACGGCTGGGTGGAATAGCGACCCTTGGCTGAGACTCAGCGGTAACTTGGAGGGACGCATGGTGTACATTGGGTTCAACATTAACTTCGTATATGAGTTCTCTAAGTTCCTCATCAAGCAGACTGCCGACGACGGGTCTACCTCCACGGAAGACATTGGGCGCTTACAGTTACGcc                                                                                                      >  1:64738/1‑301 (MQ=255)`  
                                                                                                                                                                                                    `ggAACAGACAACGATGACACATTCACTACCTCTATTCATATTCCAACAATTTATGGTGCAAACTTCGGGAGGGGAAAAATCACTGTATTGGAGCCTGATGGTGAGATAACCGTGTTTGAGCAACCTACGGCTGGGTGGAATAGCGACCCTTGGCTGAGACTCAGCGGTAACTTGGA‑GGACGCATGGTGTACGTTGGGTTCAACATTAACTTCGTATATGAGTTCTCTAAGTTCCTCATCAAGCAGACTGCCGACGACGGGTCTACCTCCACGGAAGACATTGGGCGCTTACAGTTACGCCg                                                                                                     <  1:642374/301‑1 (MQ=255)`  
                                                                                                                                                                                                       `aCATACAACGATGACACATTCACTACCTCTATTCATATTCCAACAATTTATGGTGCAAACTTCGGGAGGGGCAAAATCACTGTATTGGAGCCTGATGGTGAGATAACCGTGTTTGAGCAACCTACGGCTGGGTGGAATAGCGACCCTTTGCTGAGACTCAGCGGTAACTTGGAGGGACGCATGGTGTACATTGGGTTCAACATTAACTTCGTATATGAGTTCTCTAAGTTCCTCATCAAGCAGACTGCCGACGACGGGTCTACTTCAACGGAAGACATTGGGCGCTTACAGTTACGCCGAg                                                                                                   >  1:155344/1‑301 (MQ=255)`  
                                                                                                                                                                                                       `aCATACAACGATGACACATTCACTACCTCTATTCATATTCCAACAATTTATGGTGCAAACTTCGGGAGGGGCAAAATCACTGTATTGGAGCCTGATGGTGAGATAACCGTGTTTGAGCAACCTACGGCTGGGTGGAATAGCGACCCTTGGCTGAGACTCAGCGGTAACTTGGAGGGACGCATGGTGTACATTGGGTTCAACATTAACTTCGTATATGAGTTCTCTAAGTTCCTCATCAAGCAGACTGCCGACGACGGGTCTACCTCCACGGAAGACATTGGGCGCTTACAGTTACGCCGAg                                                                                                   >  1:75100/1‑301 (MQ=255)`  
                                                                                                                                                                                                              `aCGATGACACATTCACTACCTCTATTCATATTCCAACAATTTATGGTGCAAACTTCGGGAGGGGCAAAATCACTGTATTGGAGCCTGATGGTGAGATAACCGTGTTTGAGCAACCTACGGCTGGGTGGAATAGCGACCCTTGGCTGAGACTCAGCGGTAACTTGGAGGGACGCATGGTGTACATTGGGTTCAACATTAACTTCGTATATGAGTTCTCTAAGTTCCTCATCAAACAGACTGCCGACGACGGGTCTACCTCCACGGAAGACATTGGGCGCTTACAGTTACGCCGAGCGTgggg                                                                                            >  1:659626/1‑300 (MQ=255)`  
                                                                                                                                                                                                                `gATGACACATTCACTACCTCTATTCATATTCCAACAATTTATGGTGCAAACTTCGGGAGGGGCAAAATCACTGTATTGGAGCCTGATGGTGCGATAACCGTGTTTGAGCAACCTACGGCTGGGTGGAATAGCGACCCTTGGCTGAGACTCAGCGGTAACTTGGAGGGACGCATGGTGTACCTTGGGTTCAACATTAACTTCGTATATGAGTTCTCTAAGTTCCTCATCAAGCAGACTGCCGACTACGGGTCTACCTCCACGGAAGACATTGCGCGCTTTCCGTTACCCCGAGCGTGGGttt                                                                                          >  1:549533/1‑300 (MQ=255)`  
                                                                                                                                                                                                                `gATGACACATTCACTACCTCTATTCATATTCCAACAATTTATGGTGCAAACTTCGGGAGGGGCAAAATCACTGTATTGGAGCCTGATGGTGAGATAACCGTGTTTGAGCAACCTACGGCTGGGTGGAATAGCGACCCTTGGCTGAGACTCAGCGGTAACTTGGAGGGACGCATGGTGTACATTGGGTTCAACATTCACTTCGTATATGAGTTCTCTAAGGTCCTCATCAAGCAGACTGCCGACGACGGGTCTACCTCCCCGGAAGACATTGGGCGCTTCCAGTTACGCCGAGCGTGGGTTa                                                                                          >  1:278681/1‑301 (MQ=255)`  
                                                                                                                                                                                                                `gATGACACATTCACTACCTCTATTCATATTCCAACAATTTATGGCGCAAACTTCGGGAGGGGCAAAATCACTGTATTGGAGCCTGATGGTGAGATAACCGTGTTTGAGCAACCTACGGATGGGTGGAATAGCGACCCTTGGCTGAGACTCAGCGGTAACTTGGAGGGACGCATGGTGTACATTGGGTTCAACATTAACTTCGTATATGAGTTCTCTAAGTTCCTCATCAAGCAGACTGCCGACGACGGGTCTACCTCCACGGAAGACATTGGGCGCTTACAGTTACGCCGAGCGTGGGTTa                                                                                          >  1:249731/1‑301 (MQ=255)`  
                                                                                                                                                                                                                  `ggACACATTCACTACCTCTATTCATATTCCAACAATTTATGGTGCAAACTTCGGGAGGGGCAAAATCACTGTATTGGAGCCTGATGGTGAGATAACCGTGTTTGAGCAACCTACGGCTGGGTGGAATAGCGACCCTTGGCTGAGACTCAGCGGTAACTTGGAGGGACGCATGGTGTACATTGGGTTCAACATTAACTTCGTATATGAGTTCTCTAAGTTCCTCATCAAGCAGACTGCCGACGACGGGTCTACCTCCACGGAAGACATTGGGCGCTTACAGTTACGCCGAGCGTGGGTTAAc                                                                                        <  1:622344/300‑1 (MQ=255)`  
                                                                                                                                                                                                                   `gACACATTCACTACCTCTATTCATATTCCAACAATTTATGGTGCAAACTTCGGGAGGGGCAAAATCACTGTATTGGAGCCTGATGGTGAGATAACCGTGTTTGAGCAACCTACGGCTGGGTGGAATAGCGACCCTTGGCTGAGACTCAGCGGTAACTTGGAGGGACGCATGGTGTACATTGGGTTCAACATTAACTTCGTATATGAGTTCTCTAAGTTCCTCATCAAGCAGACTGCCGACGACGGGTCTACCTCCACGGAAGCCATAGGGCGCTTACAGTTACGCCGAGCGTGGGTTAACt                                                                                       >  1:408810/1‑301 (MQ=255)`  
                                                                                                                                                                                                                   `gACACATTCACTACCTCTATTCATATTCCAACAATTTATGGTGCAAACTTCGGGAGGGGCAAAATCACTGTATTGGAGCCTGATGGTGAGATAACCGTGTTTGAGCAACCTACGGCTGGGTGGAATAGCGACCCTTGGCTGAGACTCAGCGGT‑ACTTGGAGGGACGCATGGTGTACATTGGGTTCAACATTAACTTCGTATATGAGTTCTCTAAGTTCCTCATCAAGCAGACTGCCGACGACGGGTCTACCTCCACGGAAGACATTGGGCGCTTACAGTTACGCCGAGCGTGGGTTAACTa                                                                                      >  1:364199/1‑301 (MQ=255)`  
                                                                                                                                                                                                                    `agggcagcccTACCTCTATTCATTTTCCAACAATTTATGGTGCAAACTTCGGGAGGGGCACAACCAATGTATTGGAGCCTGTTTGTGCGATAACCGTGTTTGAGCAACCTACGGCTGGGTGGAATAGCGACGCTTGGCGGAGACTCAGCGGTAACTTGGAGGGACGCATGGTGTACATTGGGTTCAACATTAACTTCGTATATGAGTTCTCTAAGTTCCTCATCAAGCAGACTGCCGACGACGGGTCTACCTCCACGGAAGACATTGGGCGCTTACAGTTACGCCGAGCGTGGGTTAACTa                                                                                      <  1:375/292‑1 (MQ=255)`  
                                                                                                                                                                                                                           `cACTACCTCTATTCATATTCCAACAATTTATGGTGCAAACTTCGGGATGGGCAAAATCACTGTATTGGAGCCTGATGGTGAGATAACCGTGTTTGAGCAACCTACGGCTGGGTGGAATAGCGACCCTTGGCTGAGACTCAGCGGTAACTTGGAGGGACGCATGGTGTACATTGGGTTCAACATTAACTTCGTATATGAGTTCTCTAAGTTCCTCATCAAGCAGACTGCCGACGACGGGTCTACCTCCACGGAAGACATTGGGCGCTTACAGTTACGCCGAGCGTGGGTTAACTACGAGAAc                                                                               >  1:512879/1‑301 (MQ=255)`  
                                                                                                                                                                                                                           `cACTACCTCTATTCATATTCCAACAATTTATGGTGCAAACTTCGGGAGGGGCAAAATCACTGTATTGGAGCCTGATGGTGAGATAACCGTGTTTGAGCAACCTACGGCTGGGTGGAATAGCGACCCTTGGCTGAGACTCAGCGGTAACTTGGAGGGACGCATGGTGTACATTGGGTTCAACATTAACTTCGTATATGAGTTCTCTAAGTTCCTCATCAAGCAGACTGCCGACGACGGGTCTACCTCCACGGAAGACATTGGGCGCTTACAGTTACGCCGAGCGTgggctgtctcttataca                                                                               >  1:288811/1‑287 (MQ=255)`  
                                                                                                                                                                                                                               `aCCTCTATTCATATTCCAACAATTTATGGTGCAAACTTCGGGAGGGGCAAAATCACTGTATTGGAGCCTGATGGTGAGATAACCGTGTTTGAGCAACCTACGGCTGGGTGGAATAGCGACCCTTGGCTGATACTCAGCGGTAACTTGGAGGGACGCATGGTGTACATTGGGTTCAACATTAACTTCGTATATGAGTTCTCTAAGTTCCTCATCAAGCAGACTTCCGACGACGGGTCTACCTCCACGGAAGACATTGGGCGCTTACAGTTACGCCGAGCGTGGGTTCACTAAGAGAACTCTg                                                                           >  1:324752/1‑301 (MQ=255)`  
                                                                                                                                                                                                                                   `ctATTCATATTCCAACAATTTATGGTGCAAACTTCGGGAGGGGCAAAATCACTGTATTGGAGCCTGATGGTGAGATAACCGTGTTTGAGCAACCTACGGCTGGGTGGAATAGCGACCCTTGGCTGAGACTCAGCGGTAACTTGGAGGGACGCATGGTGTACATTGGGTTCAACATTAACTTCGTATATGAGTTCTCTAAGTTCCTCATCAAGCAGACTGCCGACGACGGGTCTACCTCCACGGAAGACATTGGGCGCTTACAGTTACTCCGAGCGTTGGTTAACTACGATACCTCTGGTat                                                                       >  1:665350/1‑300 (MQ=255)`  
                                                                                                                                                                                                                                   `ctATTCATATTCCAACAATTTATGGTGCAAACTTCGGGAGGGGCAAAATCACTGTATTGGAGCCTGATGGTGAGATAACCGTGTTTGAGCAACCTACGGCTGGGTGGAATAGCGACCCTTGGCTGAGACTCAGCGGTAACTTGGAGGGACGCATGGTGTACATTGGGTTCAACATTAACTTCGTATATGAGTTCTCTAAGTTCCTCATCAAGCAGACTGCCGACGACGGGTCTACCTCCACGGAAGACATTGGGCGCTTACAGTTACGCCGACCGTGGGTTAACTACGAGAACTCTGGTAc                                                                       >  1:114458/1‑301 (MQ=255)`  
                                                                                                                                                                                                                                   `ctATTCATATTCCAACAATTTATGGTGCAAACTTCGGGAGGGGCAAAATCACTGTATTGGAGCCTGATGGTGAGATAACCGTGTTTGAGCAACCTACGGCTGGGTGGAATAGCGACCCTTGGCTGAGACTCAGCGGTAACTTGGAGGGACGCATGGTGTACATTGGGTTCAACATTAACTTCGTATATGAGTTCTCTAAGTTCCTCATCAAGCAGACAGCCGACGACGGGTCTACCTCCACGGAAGACATTGGGCGCTTACAGTTACGCCGAGCGTGGGTTAAcctgtctcttatacacat                                                                       >  1:49934/1‑284 (MQ=255)`  
                                                                                                                                                                                                                                   `ctATTCATATTCCAACAATTTATGGTGCAAACTTCGGGAGGGGCAAAATCACTGTATTGGAGCCTGATGGTGAGATAACCGTGTTGGAGCAACCTACGGCTGGGTGGAATAGCGACCCTTGGCTGAGACTCAGCGGTAACTTGGAGGGACGCATGGTGTACATTGGGTTCAACATTAACTTCGTATATGAGTTCTCTAAGTTCCTCATCAAGCAGACTGCCGACGACGGGTCTACCTCCACGGAAGACATTGGGCGCTTACAGTTACGCCGAGCGTGGGTTAACTACGAGAACTCTGGTAc                                                                       <  1:174035/301‑1 (MQ=255)`  
                                                                                                                                                                                                                                     `aTTCATATTCCAACAATTTATGGTGCAAACTTCGGGAGGGGCAAAATCACTGTATTGGAGCCTGATGGTGAGATAACCGTGTTTGAGCAACCTACGGCTGGGTGGAATAGCGACCCTTGGCTGAGACTCAGCGGTAACTTGGAGGGACGCATGGTGTACATTGGGTTCAACATTAACTTCGTATATGAGTTCTCTAAGTTCCTCATCAAGCAGACTGCCGACGACGGGTCTACCTCCACGGAAGACATTGGGCGCTTACAGTTACGCCGAGCGTGGGTTAACTACGAGAACTCTGGTAcct                                                                     >  1:241008/1‑299 (MQ=255)`  
                                                                                                                                                                                                                                                 `acaaTTTATGGTGCAAACTTCGGGAGGGGCAAAATCACTGTATTGGAGCCTGATGGTGAGATAACCGTGTTTGAGCAACCTACGGCTGGGTGGAATAGCGACCCTTGGCTGAGACTCCGCGGTAACTTGGAGGGACGCATGGTGTACATTGGGTTCAACATTAACTTCGTATATGAGTTCTCTAAGTTCCTCATCAAGCAGACTGCCGACGACGGGTCTACCTCCACGGAAGACATTGGGCGCTTACAGTTACACCGAGCGTGGGTTAACTACGAGAACTCTGGTACGTTTGACATTTATg                                                         >  1:204661/1‑301 (MQ=255)`  
                                                                                                                                                                                                                                                  `cgaTTTATGGTGCAAACTTCGGGAGGGGCAAAAGCACTGTATTGGAGCCTGATGGTGAGATAACCGTGTTTGAGCAACCTACGGCTGGGGGGAATAGCGACCCTTGGCTGAGACTCAGCGGTAACTTGGAGGGACGCATGGTGTACATTGGGTTCAACATTAACTTCGTATATGAGTTCTCTAAGTTCCTCATCAAGCAGACTGCCGACGACGGGTCTACCTCCACGGAAGACATTGGGCGCTTACAGTTACGCCGAGCGTGGGTTAACTACGAGAACTCTGGTACGTTTGACATTTATGt                                                        <  1:225644/299‑1 (MQ=255)`  
                                                                                                                                                                                                                                                  `caaTTTATGGTGCAAACTTCGGGAGGGGCAAAATCACTGTATTGGAGCCTGATGGTGAGATAACCGTGTTTGAGCAACCTACGGCTGGGTGGAATAGCGACCCTTGGCTGAGACTCAGCGGTAACTTGGAGGGACGCATGGTGTACATTGGGTTCAACATTAACTTCGTATATGAGTTCTCTAAGTTCCTCCTCAAGCAGACTGCCGACGACCGGTCTACCTCCACTGAAGACATTGGGCGCTTACTGTTACGCCGCGCGTGGGTTAACTACTAGTACTCTGGTACGTTTGACATTTATGt                                                        >  1:426016/1‑301 (MQ=255)`  
                                                                                                                                                                                                                                                  `aaaTTTATGGTGCAAACTTTGGGATGTGCAAAAGCACTGTATTGGAGCCTGATGGTGAGATAACCGTGATTGAGCAACCTACGGATGGGTGGAATAGCGACCCTTGGCTGAGACTCAGCGGTAACTTGGAGCGACGCATGGTGTACATTGGGTTCAACATTAACTTCGTATATGAGTTCTCTAAGTTCCTCATCAAGCAGACTGCCGACGACGGGTCTACCTCCACGGAAGACATTGGGCGCTTACAGTTACGCCGAGCGTGGGTTAACTACGAGAACTCTGGTACGTTTGACATTTATGt                                                        <  1:43367/300‑1 (MQ=255)`  
                                                                                                                                                                                                                                                           `gTGCAAACTTCGGGAGGGGCAAAATCACTGTATTGGAGCCTGATGGTGAGATAACCGTGTTTGAGCAACCTACGGCTGGGTGGAATAGCGACCCTTGGCTGAGACTCAGCGGTAACTTGGAGGGACGCATGGTGTACATTGGGTTCAACATTAACTTCGTATATGAGTTCTCTAAGTTCCTCATCAAGCAGACTGCCGACGACGGGTCTACCTCCACGGAAGACATTGGGCGCTTACCGTTACGCCGAGCGTGGGTTAACTACGAGAACTCTGGTACGTTTGACATTTCTGTTGAGAACCa                                               >  1:468154/1‑301 (MQ=255)`  
                                                                                                                                                                                                                                                           `gTGCAAACTTCGGGAGGGGCAAAATCACTGTATTGGAGCCTGATGGTGAGATAACCGTGTTTGAGCAACCTACGGCTGGGTGGAATAGCGACCCTTGGCTGAGACTCAGCGGTAACTTGGAGGGACGCATGGTGTACATTGGGTTCAACATTAACTTCGTATATGAGTTCTCTAAGTTCCTCATCAAGCAGACTGCCGACGACGGGTCTACCTCCACGGAAGACATTGGGCGCTTACAGTTACGCCGAGCGTGGGTTAACTACGAGAACTCTGGTActgtctcttatacacatctccgagc                                               >  1:487603/1‑277 (MQ=255)`  
                                                                                                                                                                                                                                                              `cAAACTTCGGGAGGGGCAAAATCACTGTATTGGAGCCTGATGGTAAGATAACCGTGTTTGAGCAACCTACGGCTGGGTGGAATAGCGACCCTTGGCTGAGACTCAGCGGTAACTTGGAGGGACGCATGGTGTACATTGGGTTCAACATTAACTTCGTATATGAGTTCTCTAAGTTCCTCATCAAGCAGACTGCCGACGACGGGTCTACCTCCACGGAAGACATTGGGCGCTTACAGTTACGCCGAGCGTGGGTTAACTACGAGAACTCTGGTACGTTTGACATTTATGTTGAGAACCAATc                                            <  1:544577/301‑1 (MQ=255)`  
                                                                                                                                                                                                                                                                `aaCTTTGGGAGGGGCAAAATCACTGTATTGGAGCCTGATGGTGAGATAACCGTGTTTGAGCAACCTACGGCTGGGTGGAATAGCGACCCTTGGCTGAGTCTCAGCGGTAACTTGGAGGTACGCATGGAGTACATTGGGTTCAACATTAACTTCGTATATGAGTTCTCTAAGTTCCTCATCAAGCAGACTGCCGACGACGGGTCTACCTCCACGGAAGACATTGGGCGCTTACAGTTACGCCGAGCGTGGGTTAACTACGAGAACTCTGGTACGTTTGACATTTATGTTGAGAACCAATctt                                          >  1:122108/1‑299 (MQ=255)`  
                                                                                                                                                                                                                                                                `aaCTTCGGGAGGGGCAAAATCACTGTATTGGAGCCTGATGGTGAGATAACCGTGTTTGAGCAACCTACGGCTGGGTGGAATAGCGACCCTTGGCTGAGACTCAGCGGTAACTTGGAGGGACGAATGGTGTACATTGGGTTCAACATTAACTTCGTATATGAGTTCTCTAAGTTCCTCATCAAGCAGACTGCCGACGACGGGTCTACCTCCACGGAAGACATTGGGCGCTTACAGTTACGCCGAGCGTGGGTTAACTACGAGAACTCTGGTACGTTTGACATTTATGTTGAGAACCAATCGt                                          >  1:352379/1‑301 (MQ=255)`  
                                                                                                                                                                                                                                                                  `cTTCGGGAGGGGCAAAATCACTGTATTGGAGCCTGATGGTGAGATAACCGTGTTTGAGCAACCTACGGCTGGGTGGAATAGCGACCCTTGGCTGAGACTCAGCGGTAACTTGGAGGGACGCATGGTGTACATTGGGTTCAACATTAACTTCGTATATGAGTTCTCTAAGTTCCTCATCAAGCAGACTGCCGACGACGGGTCTACCTCCACGGAAGACATTGGGCGCTTACAGTTACGCCGAGCGTGGGTTAACTACGAGAACTCTGGTACGTTTGACATTTATGtctgtctcttatacaca                                        >  1:614158/1‑285 (MQ=255)`  
                                                                                                                                                                                                                                                                           `gtgCAAAATCACTGTATTGGAGCCTGATGGTGAGATAACCGTGTTTGAGCAACCTACGGCTGGGTGGAATAGCGACCCTTGGCTGAGACTCAGCGGTAACTTGGAGGGACGCATGGTGTACATTGGGTTCAACATTAACTTCGTATATGAGTTCTCTAAGTTCCTCATCAAGCAGACTGCCGACGACGGGTCTACCTCCACGGAAGACATTGGGCGCTTACAGTTACGCCGAGCGTGGGTTAACTACGAGAACTCTGGTACGTTTGACATTTATGTTGAGAACAAATCGTCTAACTGGAAg                               <  1:107490/299‑1 (MQ=255)`  
                                                                                                                                                                                                                                                                           `gggCAAAATCACTGTATTGGAGCCTGATGGTGAGATAACCGTGTTTGAGCAACCTACGGCTGGGTGGAATAGCGACCCTTGGCTGAGACTCAGCGGTAACTTGGAGGGACGCATGGTGTACATTGGGTTCAACATTAACTTCGTATATGAGTTCTCTAAGTTCCTCATCAAGCAGACTGCCGACGACGGGTCTACCTCCACGGAAGACATTGGGCGCTTACAGTTACGCCGAGCGTGGGTTAACTACGAGAACTCTGGTACGTTTGACATTTATGTTGAGAACCAATCGTCTAACTGGAAg                               <  1:592315/301‑1 (MQ=255)`  
                                                                                                                                                                                                                                                                            `ggCAAAATCACTGTATTGGAGCCTGATGGTGAGATAACCGTGTTTGAGCAACCTACGGCTGGGTGGAATAGCGACCCTTGGCTGAGACTCAGCGGTAACTTGGAGGGACGCATGGTGTACATTGGGTTCAACATTAACTTCGTATATGAGTTCTCTAAGTTCCTCATCCAGCAGACTTCCGACGACGGGTCTACCTCCACGGAAGACATTGGGCGCTTACAGTTACGCCGAGCGTGGGTTAACTACGAGAACTCTGGTACGTTTGACATTTATGTTGAGAACCAATCGTCTAACTGGAAGt                              >  1:245262/1‑301 (MQ=255)`  
                                                                                                                                                                                                                                                                              `aaaaaTCACTGTATTGGAGCCTGATGGTGAGATAACCGTGTTTGAGCAACCTACGGCTGGGTGGAATAGCGACCCTTGGCTGAGACTCAGCGGTAACTTTGAGGGACGCATGGTGTACATTGGGTTCAACATTAACCTCGTATATGAGTTCTCTAAGTTCCTCATCAAGCAGACTGCCGACGACGGGTCTACCTCCACGGAAGACATTGGGCGCTTACAGTTACGCCGAGCGTGGGTTAACTACGAGAACTCTGGTACGTTTGACATTTATGTTGAGAACCAATCGTCTAACTGGAAGTac                            <  1:555901/300‑1 (MQ=255)`  
                                                                                                                                                                                                                                                                                     `gcTGTATTGGAGGCTGATGGTGAGATAACCGGGTTTGAGCAACCTACGGCTGGGTGGAATAGCGACCCTTGGCTGAGACTCAGCGGTAACTTGGAGGGACGCATGGTGTACATTGGGTTCAACATTAACTTCGTATATGAGTTCTCTAAGTTCCTCATCAAGCAGACTGCCGACGACGGGTCTACCTCCACGGAAGACATTGGGCGCTTACAGTTACGCCGAGCGTGGGTTAACTACGAGAACTCTGGTACGTTTGACATTTATGTTGAGAACCAATCGTCTAACTGGAAGTACACAATgg                     <  1:210582/300‑1 (MQ=255)`  
                                                                                                                                                                                                                                                                                      `aggTATTGGAGCCTGATGGTGAGATAACCGTGTTTGAGCAACCTACGGCTGGGTGTAATAGCGACCCTTGGCTGAGACTCAGCGGTAACTTGGAGGGACGCATGGTGTACATTGGGTTCAACATTAACTTCGTATATGAGTTCTCTAAGTTCCTCATCAAGCAGACTGCCGACGACGGGTCTACCTCCACGGAAGACATTGGGCGCTTACAGTTACGCCGAGCGTGGGTTAACTACGAGAACTCTGGTACGTTTGACATTTATGTTGAGAACCAATCGTCTAACTGGAAGTACACAATGGc                    <  1:568802/299‑1 (MQ=255)`  
                                                                                                                                                                                                                                                                                               `aGCCTGATGGTGAGATAACCGTGTTTGAGCAACCTACGGCTGGGTGGAATAGCGACCCTTGGCTGAGACTCAGCGGTAACTTGGAGGGACGCATGGTGTACATTGGGTTCAACATTAACTTCGTATATGCGTGCTCTAAGTTCCTCATCAAGCAGACTGCCGACGACGGGTCTACCTCCACGGAAGACATTGGGCGCTTACAGTTACGCCGAGCGTGGGCTAACTACGAGAACTCTGGTACGATTGACATTTATGTTGAGAACCAATCGTCTAACTGGCAGTACACAATGGCTGGTGccct           >  1:44045/1‑300 (MQ=255)`  
                                                                                                                                                                                                                                                                                                 `ccTGATGGTGAGATAACCGTGTTTGAGCAACCTACGGATGGGTGGAATAGCGACCCTTGGCTGAGACTCAGCGGTAACTTGGAGGGACGCATGGTGTACATTGGGTTCAACATTAACTTCGTATATGAGTTCTCTAAGTTCCTCATCAAGCAGACTGCCGACGACGGGTCTACCTCCACGGAAGACATTGGGCGCTTACAGTTACGCCGAGCGTGGGTTAACTACGAGAACTCTGGTACGTTTGACATTTATGTTGAGAACCAATCGTCTAACTGTAAGTACACAATGGCTGGTGCCCGAt         <  1:580092/301‑1 (MQ=255)`  

                                                                                                                                                                                                                                                                                                          `|`                                                                                                                                                                                                                                                                                                            
`GTGGTCTCATTGGGACTTTGGGGAAAACGTACAGGTTCTAGCTTGTCAGAGTATCAGCTCAGATATGTATGTGATTCTTCGCAATGAGTTCAATACGTTCCTAGCTAGAATCTCTTTCACTAAGAACGCCATTGACTTACAGGGAGAACCCTATCGTGCCTTTATGGACATGAAGATTCGATACACGATTCCTAGTGGAACATACAACGATGACACATTCACTACCTCTATTCATATTCCAACAATTTATGGTGCAAACTTCGGGAGGGGCAAAATCACTGTATTGGAGCCTGATGGTAAGATAACCGTGTTTGAGCAACCTACGGCTGGGTGGAATAGCGACCCTTGGCTGAGACTCAGCGGTAACTTGGAGGGACGCATGGTGTACATTGGGTTCAACATTAACTTCGTATATGAGTTCTCTAAGTTCCTCATCAAGCAGACTGCCGACGACGGGTCTACCTCCACGGAAGACATTGGGCGCTTACAGTTACGCCGAGCGTGGGTTAACTACGAGAACTCTGGTACGTTTGACATTTATGTTGAGAACCAATCGTCTAACTGGAAGTACACAATGGCTGGTGCCCGATTAGGCTC  >  NC_001604.1/26458‑27054`

  

Alignment Legend

Aligned base mismatch/match (shaded by quality score): `ATCG/ATCG` `< 3 ≤` `ATCG/ATCG` `< 11 ≤` `ATCG/ATCG` `< 23 ≤` `ATCG/ATCG` `< 34 ≤` `ATCG/ATCG` `< 38 ≤` `ATCG/ATCG`

Unaligned base: `atcg`    Masked matching base: `atcg`    Alignment gap: `‑`    Deleted base: `‑`
